# Supplementary material for: The Influence of the Mechanical Compliance of a Substrate on the Morphology of Nanoporous Gold Thin Films
Source: Nanomaterials (Basel). 2024 Apr 25;14(9):758. doi: 10.3390/nano14090758 (PMC11085319; doi:10.3390/nano14090758)
Supplement: Supplementary file 1 [file nanomaterials-14-00758-s001.zip › nanomaterials-2977853-supplementary.pdf]

Supplementary Materials

Influence of Mechanical Compliance of the Substrate on the Morphology of Nanoporous Gold Thin Films

Sadi Shahriar [1], Kavya Somayajula [2], Conner Winkeljohn [1], Jeremy K. Mason[1] and Erkin Seker [3,*]

[1] Department of Materials Science and Engineering, University of California - Davis, Davis, CA 95616, USA

[2] Department of Mechanical and Aerospace Engineering, University of California - Davis, Davis, CA 95616, USA

[3] Department of Electrical and Computer Engineering, University of California - Davis, Davis, CA 95616, USA

* Correspondence: eseker@ucdavis.edu; Tel.: +1-(530)-752-7300

S1. SEM Image Analysis for Characterizing the Morphological Features

S1.1. Analysis of the Discrete Islands Bound by Macroscopic Cracks

Edge-detection and gain extract in GIMP were applied to 8-bit grayscale images to accentuate the cracks against the background. The Sauvola auto local threshold method was applied to the GIMP-processed image to obtain binary segmented images separating the cracks from the background, with the cracks and the areas inside the cracks given pixel values of 0 and 255, respectively. An erosion process was applied to the binary images to close the boundaries of the discrete islands. Finally, the elliptical outlines of the closed regions were obtained, and the major and minor axes lengths of the ellipses were measured in Fiji. Figure S1 shows representative images of these processing steps.

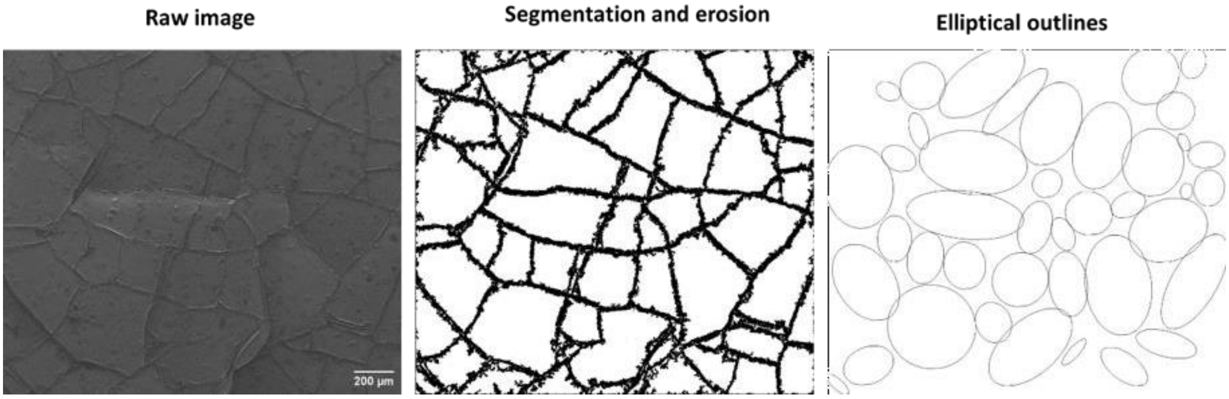

Figure S1. The process of obtaining elliptical outlines of the discrete islands bound by macroscopic cracks.

S1.2. Analysis of the Microscopic Cracks

8-bit images were subjected to trainable Weka segmentation in Fiji to obtain binary images such that the cracks (pixel value of 255) from the background (pixel value of 0). A size threshold of 30 pixels was then applied to filter out the smaller specks that were not cracks. The area of each crack was computed in Fiji to obtain the average crack area in an image. The total crack area in an image was divided by the image area to calculate the crack coverage percentage. Figure S2 shows a raw SEM image and the corresponding segmented image showing the cracks in black against a white background.

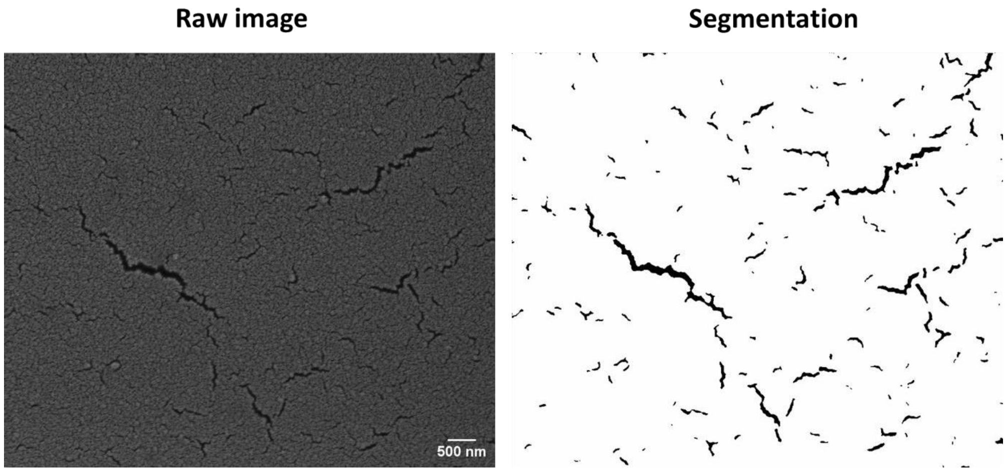

Figure S2. Binary image obtained from a raw image of the microscopic cracks through Weka segmentation.

S1.3. Analysis of the Ligament and Pore Widths

8-bit images were subjected to Otsu local auto threshold to turn the images into binary images where pores turn into pure black (0) and the ligaments turn into pure white (255) pixels. The segmentation was followed by the application of a median filter to smoothen the edges of the morphological features. The resulting binary image was considered as a matrix of pixel values consisting of 0 and 255. A chord length distribution (CLD) approach was used to measure the ligament and pore widths where a chord is a line segment with each of its interior points located in one of the two phases (ligament or pore) and the end points touching the interfaces with the other phase. To find the CLD, we used a custom MATLAB script which scans pixel-by-pixel along each row and column of the matrix and records whenever it encounters the pixel value of the desired phase. For example, after the code starts running, each time the algorithm detects a pixel value of 0, it records it as one of the end points of a pore chord and stops assigning pixels to that chord after a value of 255 is encountered. This identifies the chord of a specific length corresponding to a pore and after scanning along every row, all the horizontally-oriented porechords were identified. Similarly, scanning along the columns provided the ligament andpore widths along the vertical axis. Figure S3 shows the binary image obtained by thresholding a raw image.

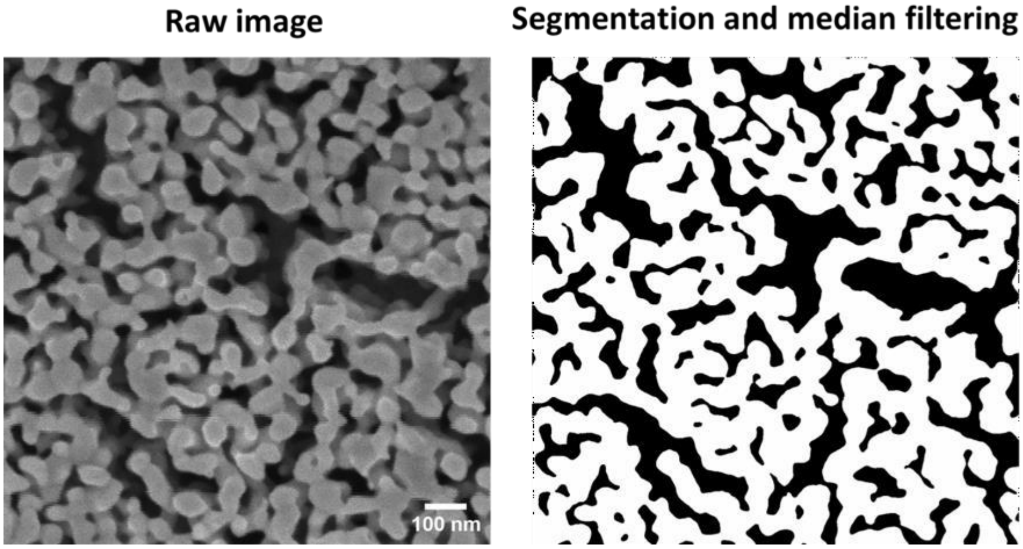

Figure S3. Binary image of ligaments and pores showing the ligaments in white and the pores in black obtained by thresholding and median filtering the raw image.

S1.4. Analysis of the Macroscopic Cracks in np-Au/Glass

The macroscopic cracks on glass do not form discrete islands and hence, cannot form elliptical outlines. Therefore, the SEM images for np-Au/glass were made binary to segment the cracks (Figure S4) and the MATLAB script for ligament and pore width extraction was applied to the binary images to compute the distances between neighboring cracks. However, distributions of distances found by the two methods do not match exactly since the definitions of crack-to-crack distance are different for the two methods. To make the crack-to-crack distances in np-Au/glass comparable with those in np-Au/PDMS computed by the elliptical outline method, the distances on glass were multiplied by a factor of 1.53. This factor was found by applying the MATLAB script to images of np-Au on 3.18 mm-thick PDMS to measure the crack-to-crack distances. Then, the ratio of the median distances by the elliptical method (~153 μm) to that obtained by the MATLAB code (~100 μm) was used as the upscaling factor for the distances on glass. The rationale for choosing the 3.18 mm-thick PDMS substrates for calculating the upscaling factor is that the density of cracks in the films supported by them is visually similar to those in theglass-supported films.

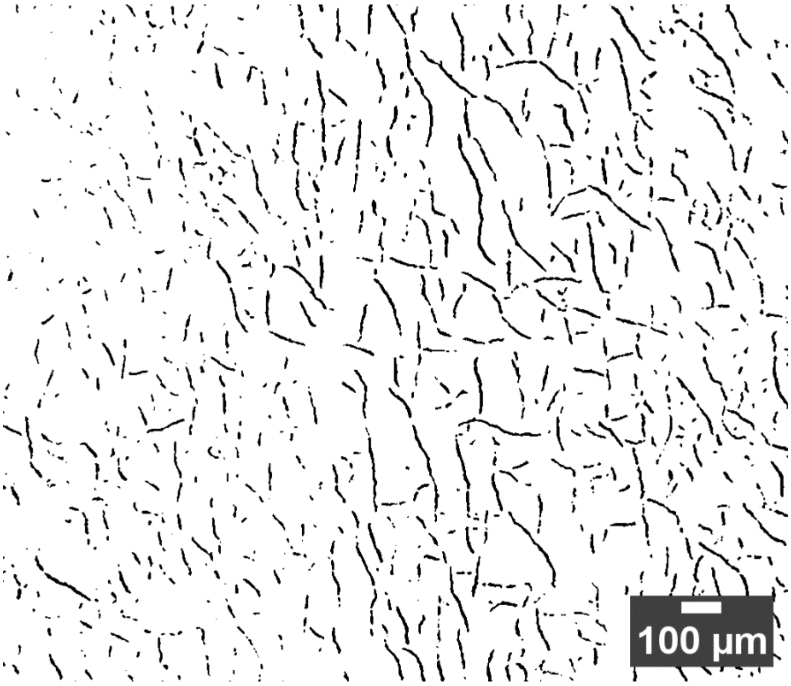

Figure S4. Binary image showing the segmentation of the macroscopic cracks in np-Au/glass at 150× magnification.

S2. Image-to-Image and Sample-to-Sample Comparison

Three images from each sample (total two samples) at different locations for each type of film-substrate combination were analyzed. Figures S5–S7 show the comparisons among the images from each sample where 'Im' denotes image. Figure S8 shows the comparison between the samples where 'S' denotes sample.

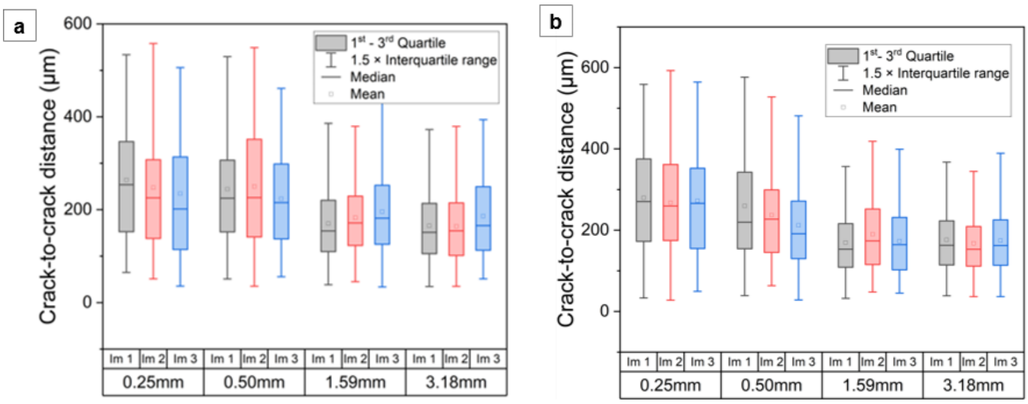

Figure S5. Macroscopic crack-to-crack distance comparison across images at different locations of (a) sample 1, and (b) sample 2.

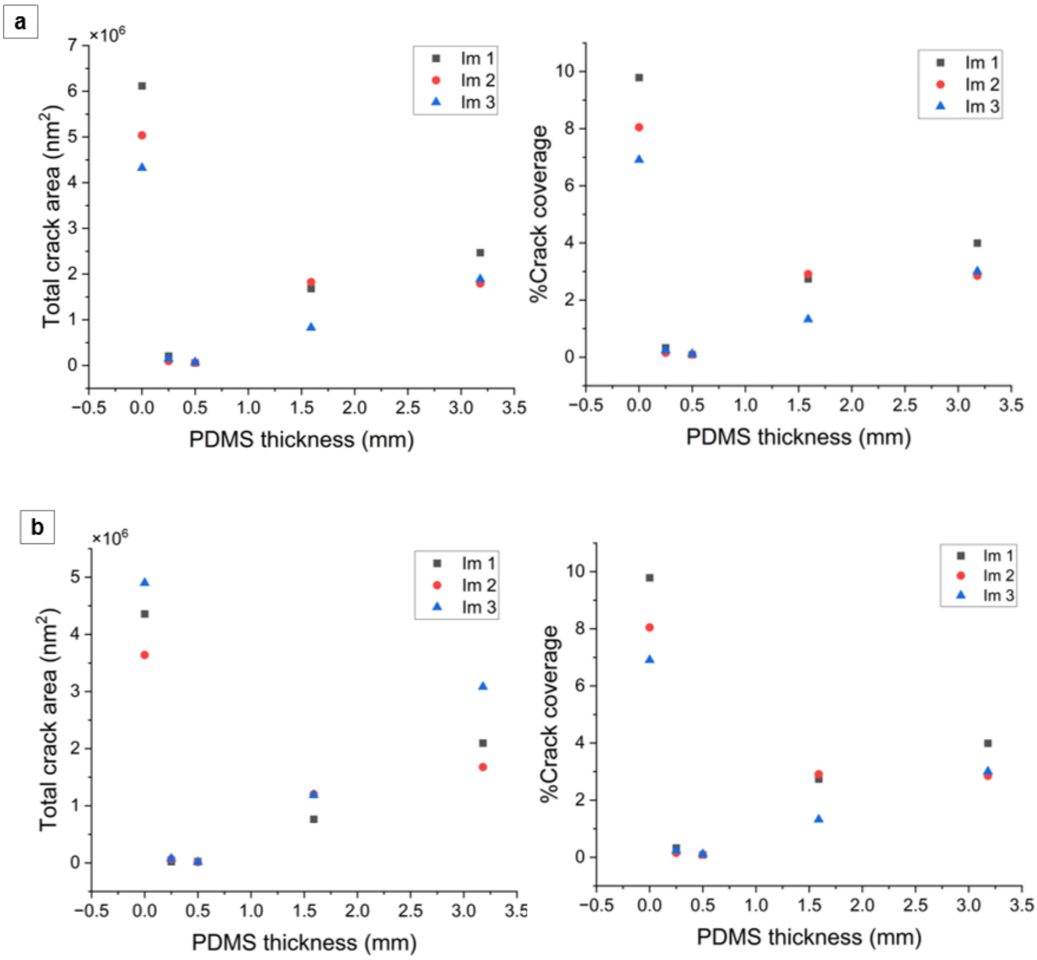

Figure S6. Total microscopic crack area and crack coverage percentage across images at different locations for (a) sample 1, and (b) sample 2.

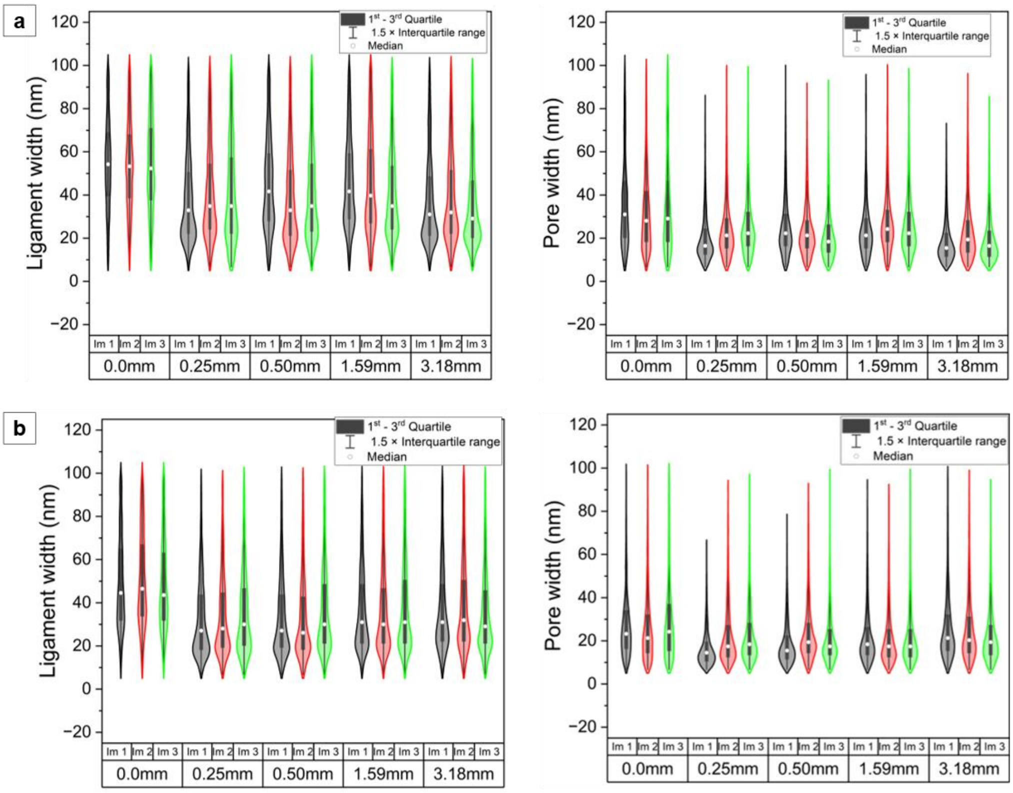

Figure S7. Ligament and pore widths distribution across images at different locations for (a) sample 1, and (b) sample 2.

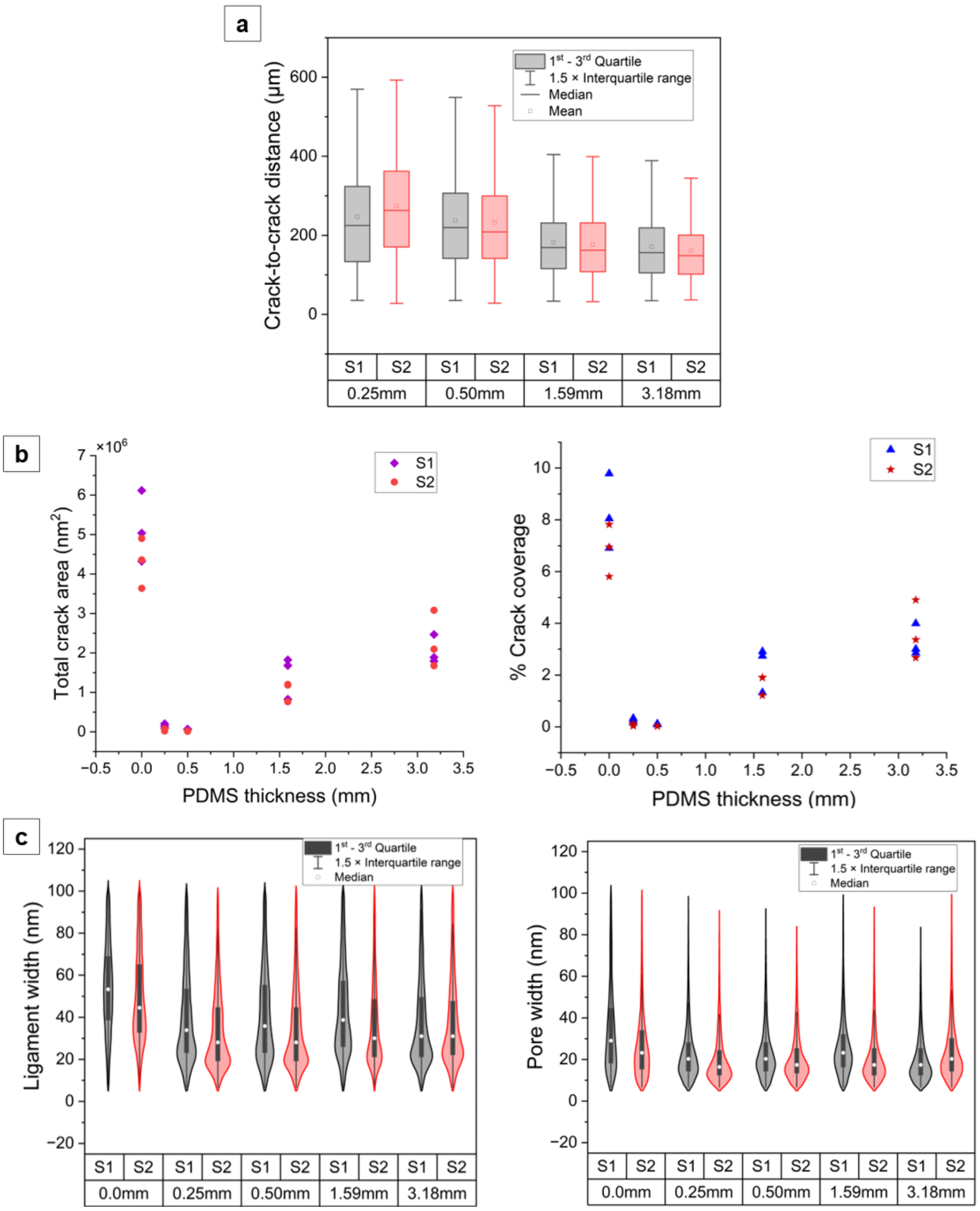

Figure S8. (a) Macroscopic crack-to-crack distance, (b) Total crack area and crack coverage percentage, and (c) ligament and pore width comparison between sample 1 and 2.

The distribution histograms of the ligament and pore widths with combined data from both samples are shown in Figure S9.

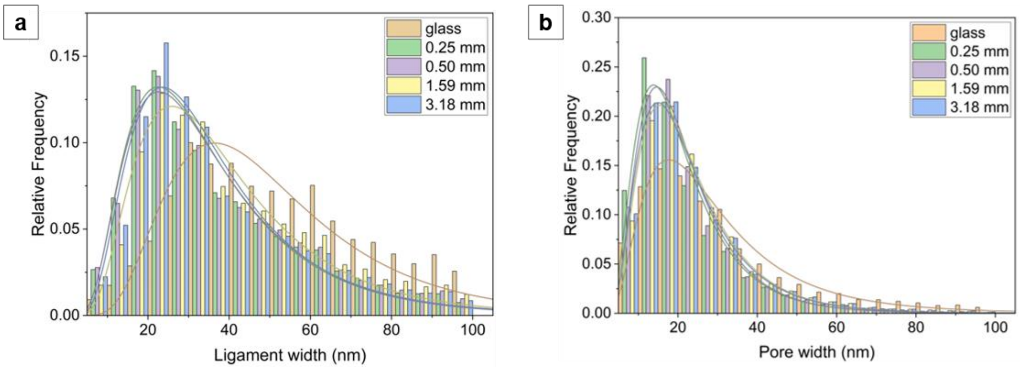

Figure S9. Distribution histograms for (a) ligament widths and (b) pore widths for glass substrate and PDMS substrates with different thicknesses.

S3. Additional Experiments with Thinner Glass Substrates and Higher Maximum PDMS Thickness

We performed an additional set of experiments where we performed 5 minutes of dealloying at room temperature followed by 15 minutes of dealloying at 55 °C for the AuAg films deposited on PDMS substrates of thicknesses 0.25, 3, and 5 mm attached to 0.20 mm-thick glass coverslips. The SEM images of the dealloying-induced microscopic cracks and plots for the distribution of crack widths are shown in Figure S10a and Figure S10b, respectively.

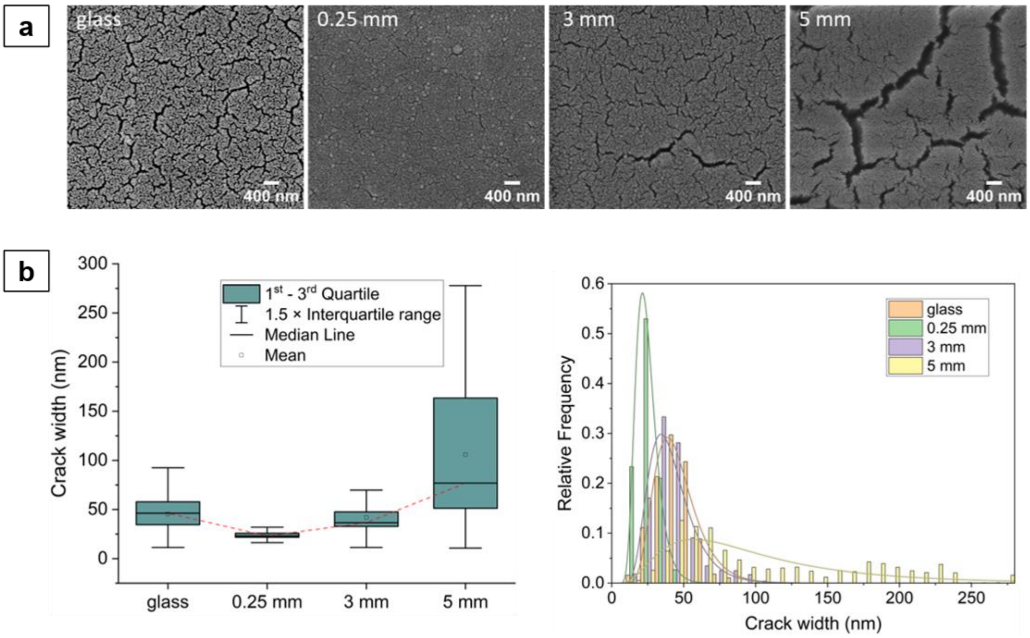

Figure S10. (a) SEM images of the microscopic cracks in np-Au/glass and np-Au/PDMS thin films, and (b) plots depicting the width distribution of the microscopic cracks. The red lines in the bar plot correspond to the median values and the error bars show the standard deviations.

Figure S11 shows tilted SEM images of np-Au films on glass and 1.59 mm-thick PDMS from the main set of experiments showing the differences in the widening of the cracks.

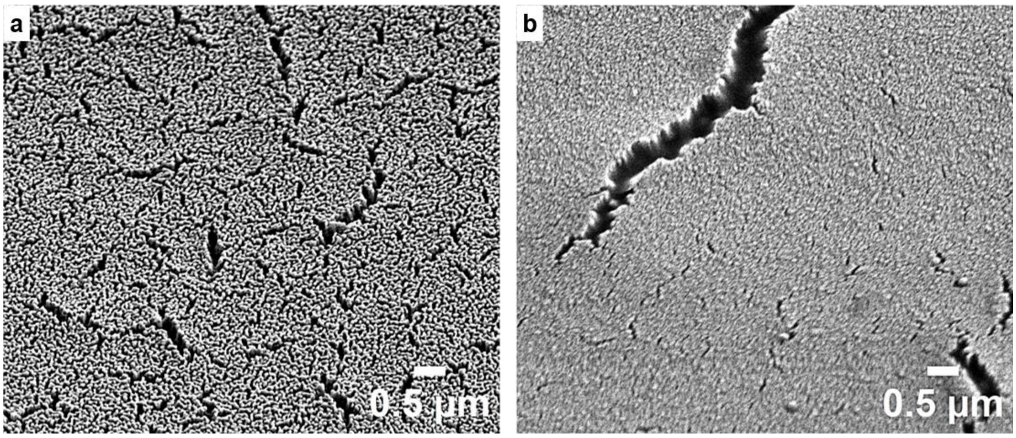

Figure S11. SEM of microscopic cracks tilted at 45° in (a) np-Au/glass film, and (b) np-Au/1.59 mm PDMS film showing the differences in crack opening in both cases.

S4. Finite-Element Simulation Methodology

Simulations were performed to estimate a) the effective elastic modulus of PDMS of variable thickness anchored to a glass substrate, b) the dependence of the strain energy of the thin film layers and the PDMS substrates after deposition and dealloying on the PDMS thickness, and c) average horizontal and vertical edge deformation magnitude at the PDMS-metal stack interface as a function of PDMS thickness. The simulations were performed using COMSOL Multiphysics software, and the experimentally-found residual stress value of ~100 MPa was used to estimate the thermal strain experienced by the AuAg, Au, and Cr films following deposition. The corresponding thermal strain was translated into a temperature difference using the coefficient of thermal expansion of the film and fed into the software as the thermal contraction condition. The simulations involved AuAg/Au/Cr layers for the post-deposition and np-Au/Au/Cr layers for the post-dealloying conditions with the PDMS substrates fixed at the bottom to mimic being anchored to rigid glass slides. For the first set of simulations the PDMS thicknesses were 0.01, 0.10, 0.15, 0.25, 0.50, 1.59, 3.18 and 5 mm, and for the second set of simulations were 0.05, 0.10, 0.15, 0.25, 0.50, 0.75, 1, 1.59, 3.18, and 5 mm. The first set of simulations was in 2D, whereas the second set was in 3D. The thickness of the AuAg and the np-Au films in the simulations were 600 nm and 500 nm, respectively. The finite element mesh for the AuAg/Au/Cr/PDMS and np-Au/Au/Cr/PDMS stacks used in the second set of simulations is shown in Figure S12.

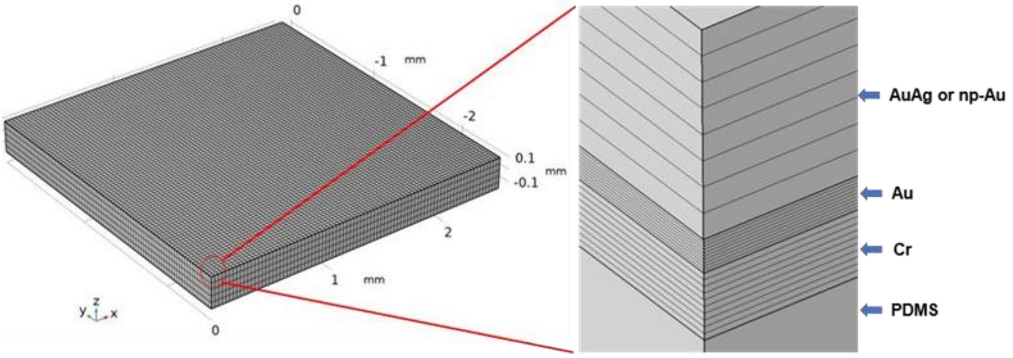

Figure S12. Finite element mesh for the post-deposition and post-dealloying film/substrate stack.

To estimate the effective surface modulus, two points on the PDMS top surface were displaced axially in the x-direction per the nominal strain values, and the effective stiffness was estimated from the force-displacement plots. The initial separation distance between two such points on the PDMS was fixed at 200 μm. To estimate the variation in strain energy upon dealloying, the thermal strains in the AuAg, Au, and Cr layers are applied as a pre-strain in the dealloying simulations.
